# Description of three new Dryocola species: Dryocola mayonis sp. nov., Dryocola sharpae sp. nov. and Dryocola baronae sp. nov., isolated from human clinical samples

**DOI:** 10.1099/ijsem.0.006986

**Published:** 2025-12-02

**Authors:** Hamidu T. Mohammed, Christina A. Koscianski, Garrett G. Gordy, Stephen Johnson, Robin Patel

**Affiliations:** 1 Division of Clinical Microbiology, Department of Laboratory Medicine and Pathology, Mayo Clinic, Rochester, MN 55905, USA; 2 Department of Quantitative Health Sciences, Mayo Clinic, Rochester, MN 55905, USA; 3 Division of Public Health, Infectious Diseases, and Occupational Medicine, Department of Medicine, Mayo Clinic, Rochester, MN 55905, USA

**Keywords:** *Dryocola*, novel species, whole-genome sequence

## Abstract

Three previously uncharacterized bacterial species, designated strains BD586^T^, BD613^T^ and BD626^T^, isolated from human clinical specimens, were identified at Mayo Clinic, Rochester, Minnesota, USA. Initial identification efforts using matrix-assisted laser desorption ionization-time-of-flight MS and partial 16S rRNA gene sequencing proved inconclusive. Comprehensive analysis involving phenotypic characterization, biochemical assays and whole-genome sequencing was undertaken. The isolates were Gram-negative, motile, facultatively anaerobic rods, occurring singly, in pairs and in short chains; BD613^T^ additionally formed small aggregates. The isolates tested positive for catalase and negative for oxidase. Their colonies appeared smooth, white, opaque and non-haemolytic. Growth was observed at 35 °C under aerobic, anaerobic and CO₂-enriched conditions, as well as in media with NaCl concentrations up to 10% and at pH 7-9. Phylogenetic relationships were inferred from core gene alignments, average nucleotide identity and digital DNA–DNA hybridization comparisons. Results confirmed the placement of BD586^T^, BD613^T^ and BD626^T^ within the recently established *Dryocola* genus, while also indicating their novelty as distinct species. The major cellular fatty acids were C_16:0_ and C_17:0_ cyclo. Based on these findings, a formal description of three new species, *Dryocola mayonis* sp. nov. (type strain BD586^T^=TSD 474^T^, =NCTC 15089^T^, =DSM 119465^T^), *Dryocola sharpae* sp. nov. (type strain BD613^T^=TSD 475^T^, =NCTC 15090^T^, =DSM 119466^T^) and *Dryocola baronae* sp. nov. (type strain BD626^T^=TSD 476^T^, =NCTC 15091^T^, =DSM 119479^T^), is proposed, and the genus description of *Dryocola* is emended to refine its genomic, morphological and chemotaxonomic boundaries.

## Introduction

The description of new species advances scientific knowledge, with each new species offering fresh perspectives on evolutionary biology, microbial ecology and pathogenicity. Furthermore, exploring the genetic and metabolic diversity of bacteria opens new avenues for understanding how microorganisms adapt to diverse environments, compete or cooperate with each other and influence the health of their hosts. Such knowledge not only enriches comprehension of the microbial world but also uncovers potential applications for disease control. Against this background, the clinical microbiology laboratory at Mayo Clinic in Rochester, Minnesota, an academic reference laboratory, has undertaken to characterize and describe bacterial isolates that failed identification using standard methods, such as matrix-assisted laser desorption ionization-time-of-flight (MALDI-ToF) MS, biochemical identification and partial 16S rRNA gene sequencing.

The family *Enterobacteriaceae* comprises 38 validly published genera bearing the correct name under the International Code of Nomenclature of Prokaryotes (ICNP), with *Escherichia* as the type genus (https://lpsn.dsmz.de/family/enterobacteriaceae; accessed on 06 September 2025) [1]. Members of this family include pathogens of humans, plants and insects, as well as environmental organisms. Genera of clinical pathogenic importance include *Cedecea*, *Citrobacter*, *Enterobacter*, *Salmonella* and *Shigella*, among others [1,7].

The clinical bacteriology laboratory at Mayo Clinic, Rochester, MN, USA, identified three bacterial isolates from three different human subjects, designated BD586^T^, BD613^T^ and BD626^T^. While MALDI-ToF MS of the described isolates yielded hits below thresholds required for identifcation, phylogenetic analysis placed the isolates in *Dryocola*, a genus described in 2023, comprising two species, *Dryocola clanedunensis* and *Dryocola boscaweniae*, isolated from rhizosphere soil of oaks, and forming a distinct monophyletic clade between the genera *Cedecea* and *Buttiauxella* [8]. Based on the application of a polyphasic approach, it is proposed that BD586^T^, BD613^T^ and BD626^T^ are three new species belonging to the genus *Dryocola* in the family *Enterobacteriaceae*.

## Methods

### Isolation of isolates

BD586^T^, BD613^T^ and BD626^T^ were isolated from clinical samples derived from the right knee, the right foot and the knee (unspecified laterality) of three distinct patients, respectively, on tryptic soy agar with 5% sheep blood incubated at 35 °C in 5–7% CO_2_.

### DNA extraction and sequencing

Procedures were performed following previously described methods [9]. Briefly, pelleted bacterial cultures (1 ml) were resuspended in 500 µl of 1X Tris/EDTA buffer at pH 8.0 and lysed with 80 µl of universal buffer solution containing lysozyme, achromopeptidase, lysostaphin and mutanolysin at 37 °C for 1 h. Following treatment with SDS and proteinase K, DNA was extracted using the Maxwell^®^ RSC Tissue DNA protocol. Extracted DNA was quantified, assessed for size and purity, sheared to 12–15 kb fragments and purified using AMPure PB beads. SMRTbell libraries were made with 500 ng DNA using the Microbial SMRTbell Express Template Prep Kit 2.0 and sequenced on a Sequel II platform as per Pacific Biosciences’ protocol.

### Proteomic and genomic characterization and phylogenomic analysis

Initial identification was performed by MALDI-ToF MS using the MALDI Biotyper^®^ Sirius platform (Bruker Scientific) and partial 16S rRNA gene sequencing with Microseq^™^ and SmartGene IDNS^™^ Centroids (SGC) analysis, according to the manufacturers’ protocols. Genomes were submitted to the type strain genome server (TYGS) for genome-relatedness analysis in the TYGS database [10, 11]. Genomes of the nearest type strains were obtained from GenBank. Genome assembly statistics were extracted using sequence-stats version 1.0 [12]. For pangenome analysis, genomes were annotated using Prokka version 1.14 [13], and Roary version 3.13.0 [14] was employed with a 70% identity threshold. The resulting core gene alignment from Roary was used to construct a maximum-likelihood phylogenetic tree with ultrafast bootstrap (1,000 replicates) in IQ-TREE [15]. TYGS-derived phylogenetic trees were built using FASTME v2.1.6.1 based on genome blast distance phylogeny (GBDP) genome distances with pseudo bootstrap support (100 replicates) [16, 17, 18]. Average nucleotide identities (ANIs) were calculated using fastANI v1.33 [19]. Digital DNA–DNA hybridization (dDDH) values were acquired from the TYGS report. Genomes were analysed for antibiotic resistance genes with AMRFinder version 3.12.8 and the AMRFinderPlus database version 24-05-02.2 [20, 21].

### Phenotypic tests

Isolates were cultured on tryptic soy agar supplemented with 5% sheep blood under aerobic conditions and supplemented with 5–7% CO₂ and on CDC anaerobic 5% sheep blood agar (Becton Dickinson) under anaerobic conditions. Gram staining was performed to assess cellular morphology. Motility was evaluated in a semi-solid medium containing 0.3% (w/v) agar. Catalase activity was assessed using 3% (v/v) H₂O₂, and oxidase activity using the drop method and oxidase test strips. Growth at different temperatures (30–37 °C) was tested in tryptic soy agar supplemented with 5% sheep blood. Growth in the presence of NaCl was tested in nutrient broth containing 0, 6 or 10% (w/v) NaCl and incubated at 35–37 °C. Growth at different pH values was tested in tryptic soy broth adjusted to pH 4–10 using acetic acid or sodium hydroxide and incubated at 35–37 °C overnight. Transmission electron microscopy was performed to assess cellular morphology, as previously described [9]. Briefly, bacteria were fixed with paraformaldehyde and glutaraldehyde, post-stained with uranyl acetate and lead citrate and imaged using a JEOL 1400+ transmission electron microscope. Flagellar staining was performed using the RYU method (Remel, USA). Fresh cultures grown on brain–heart infusion slants at 37 °C in 5–7% CO₂ were suspended in sterile distilled water, applied to glass slides, air-dried and stained for ~3 min. Slides were rinsed with deionized water, air-dried and examined under oil immersion using a Keyence BZ-X800 microscope at 1,000×total magnification. Biochemical characterization was performed using API 50 CH, API ZYM and API 20 E (bioMérieux) following the manufacturer’s instructions. Strips were incubated at 37 °C under aerobic conditions, and results were read at 4 h for API ZYM and 24 and 48 h for API 50 CH and API 20 E.

### Antimicrobial susceptibility assessment

Antimicrobial susceptibility testing was performed by the clinical microbiology laboratory, Mayo Clinic, using agar dilution, with results interpreted according to the Clinical and Laboratory Standards Institute guideline M100 [22].

### Chemotaxonomic analysis

BD586^T^, BD613^T^ and BD626^T^ were analysed by DSMZ services, Leibniz-Institut DSMZ – Deutsche Sammlung von Mikroorganismen und Zellkulturen GmbH, Braunschweig, Germany. For biomass production, strains were grown in tryptic soy agar supplemented with 5% sheep blood at 37 °C under aerobic conditions. Cellular fatty acids were converted to fatty acid methyl esters following Sasser [23]and analysed by GC-FID and GC-MS using an Agilent 7000D system [24]. Polar lipids were extracted from freeze-dried cell material of strains BD586^T^, BD613^T^, BD626^T^ and control strain, *D. boscaweniae* CCUG 76178, with a chloroform:methanol:0.3% aqueous NaCl mixture, with recovery into the chloroform phase (modified from [25], and separated by two-dimensional silica gel TLC.

## Results and discussion

### Preliminary identification of clinical isolates using MALDI-ToF MS and partial 16S rRNA gene sequencing

Initial clinical identification of isolates BD586^T^, BD613^T^ and BD626^T^ focused on using MALDI-ToF MS and partial 16S rRNA gene PCR sequencing, with the latter analysed using both Microseq^™^ and SGC. For BD586^T^, MALDI-ToF MS yielded top hits to *Buttiauxella warmboldiae* and *Buttiauxella ferragutiae* with scores of 1.65 and 1.51, respectively, while partial 16S rRNA gene sequence analysis yielded top hits to *Citrobacter koseri* and *Salmonella enterica* with 97.4–97.9% sequence identity (both Microseq and SGC). For BD613^T^, MALDI-ToF MS yielded top hits to *Buttiauxella gaviniae* with a score of 1.68, while partial 16S rRNA gene analyses yielded top hits to *S. enterica* (Microseq, 96.0% identity) and *Enterobacter mori* (SGC, 96.7% identity). For BD626^T^, the top MALDI-ToF MS hit was *B. gaviniae* with a score of 1.81, while the top 16S rRNA gene analyses hits included *Pantoea agglomerans* (Microseq, 97.9% identity) and *Enterobacter tabaci* (SGC, 98.6% identity). All MALDI-ToF MS and 16S rRNA results were below thresholds required for identification. Failure of these methods to yield definite identifications led to isolate characterization via whole-genome sequencing.

### Genome characterization and phylogenomics

Genome features of strains BD586^T^, BD613^T^ and BD626^T^, including size, sequencing coverage, N50, G+C content and gene annotations, are summarized in Table 1. Their genomes ranged from 4.3 to 5.1 Mb, with G+C contents of 54.5–56.2 mol%. All three genomes encoded a predicted CMY2/MIR/ACT/EC family class C *β*-lactamase. Additionally, BD586^T^ encoded a predicted fosfomycin resistance glutathione transferase, while BD626^T^ encoded a predicted Vat family streptogramin A *O*-acetyltransferase.

**Table 1.** General genomic features of *D. mayonis* BD586^T^, *D. sharpae* BD613^T^ and *D. baronae* BD626^T^. Genome assembly statistics were extracted using sequence-stats version 1.0 [12] and genomes were annotated using Prokka version 1.14.6 [13]

	BD586^T^	BD613^T^	BD626^T^
Genome size (bp)	4,267,658	4,424,714	5,135,068
Sequence coverage	685.6X	743.0X	423.4X
N50 value (bp)	4,267,658	4,424,714	5,028,134
G+C content (mol%)	56.21	54.55	55.85
Protein-coding sequences	4,280	4,046	4,685
rRNA	22	22	22
tRNA	84	82	85
tmRNA	1	1	1

Delineation of bacterial species is now primarily based on genome-wide metrics, with dDDH and ANI serving as the accepted standards. Thresholds of 70% for dDDH and 95% for ANI are widely used to demarcate species boundaries. Sequences from the three isolates were submitted to the TYGS platform, which identified 30 closely related type strains. The dDDH report indicated that all comparisons were below the 70% species delineation threshold, with the top dDDH score being 35.2% for BD586^T^ compared with *D. boscaweniae*, 29.5% for BD626^T^ compared with *D. boscaweniae* and 35.8% for BD613^T^ compared with *D. clanedunensis* (Tables 2 and S1, available in the online Supplementary Material). This supported the placement of the isolates within *Dryocola* but as distinct species.

**Table 2.** ANI and dDDH values for *D. mayonis* BD586^T^, *D. sharpae* BD613^T^ and *D. baronae* BD626^T^ compared with selected type strains Only the most relevant comparisons are shown here (*Dryocola* species, *C. sulfonylureivorans* and ‘*C. colo*’), which establish novelty and placement within *Dryocola*. Comparisons with complete TYGS output of 30 closest type strains are provided in Table S1. For dDDH, self-comparisons are indicated as na because TYGS does not report dDDH values for a genome against itself.

	ANI			dDDH		
Strain	BD586^T^	BD613^T^	BD626^T^	BD586^T^	BD613^T^	BD626^T^
BD586^T^	100	88.28	86.37	na	34.6	30.7
BD613^T^	88.35	100	85.66	34.6	na	29.8
BD626^T^	86.29	85.76	100	30.7	29.8	na
*D. boscaweniae* H6W4	88.62	88.64	85.73	35.2	35.8	29.5
*D. clanedunensis* H11S18	84.34	84.07	84.64	26.7	26.6	26.8
*C. sulfonylureivorans* LAM2020	84.25	84.15	84.56	27	26.6	27
‘*C. colo*’ ZA_0188	87.56	91.40	85.31	33.6	44.2	29.2

ANI comparisons against the TYGS-derived closest type strains showed that BD586^T^, BD613^T^ and BD626^T^ had the highest ANI matches with *D. boscaweniae* (88.62, 88.64 and 85.73%, respectively). The next closest matches were *D. clanedunensis* and *Cedecea sulfonylureivorans* LAM2020 with ANIs of 84.33±0.30% (Table 2). Because ‘*Cedecea colo*’ has previously been reported to cluster with *Dryocola* rather than *Cedecea* [8], dDDH and ANI analyses were rerun to include this strain. BD613^T^ showed 91.4% ANI with ‘*C. colo*’ and a dDDH value of 44.2%, while the other comparisons were lower (Table 2). Although BD613^T^ was more closely related to ‘*C. colo*’ than to any other strain tested, both ANI (91.4%) and dDDH (44.2%) values fell below accepted species-level thresholds, confirming that they are distinct species. Taken together, calculated ANI and dDDH values, all falling below species-level thresholds, support that BD586^T^, BD613^T^ and BD626^T^ are novel species within the genus *Dryocola*.

**Fig. 1.**
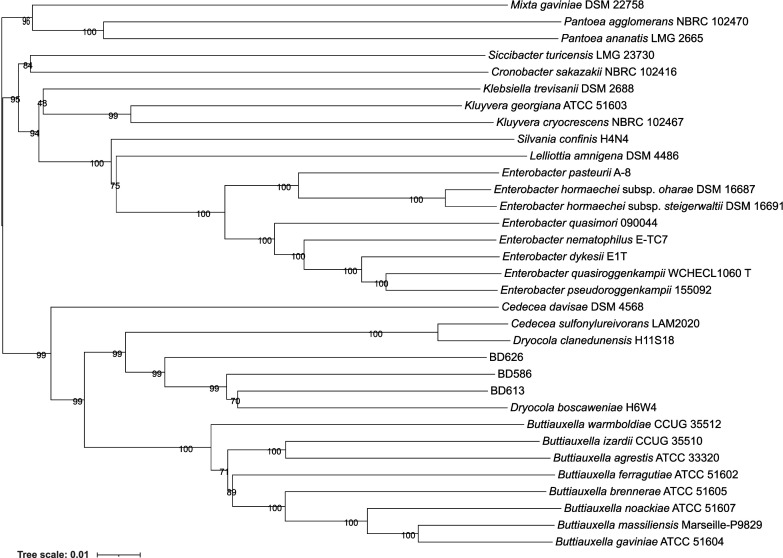
TYGS-reported phylogram from whole-genome sequences obtained from strains BD586^T^, BD613^T^, BD626^T^ and 30 closest type strains. The tree was derived with FastME 2.1.6.1 [18] and the midpoint rooted using the distance Wagner procedure [17]. Node numbers represent GBDP pseudo bootstrap support values from 100 replications.

Having established that BD586^T^, BD613^T^ and BD626^T^ represent novel species based on dDDH and ANI comparisons, their phylogenetic placement within the *Enterobacteriaceae* family was examined using TYGS-derived and core gene phylogenies. The TYGS-derived phylogenetic tree constructed using GBDP distances estimated from 16S rRNA gene sequences showed low reliability (pseudo bootstrap support of 50.8%) and was therefore not further considered (data not shown). However, the TYGS tree constructed from the GBDP distances from whole-genome sequences provided robust support (pseudo bootstrap support of 93.9%) for the established phylogenetic relationships (Fig. 1). Here, BD586^T^, BD613^T^ and BD626^T^ clustered with *D. clanedunensis*, *D. boscaweniae* and *C. sulfonylureivorans*, forming a branch with 99% bootstrap support. This *Dryocola*–*C. sulfonylureivorans* clade, together with *Buttiauxella*, was positioned as a sister lineage to *Cedecea*, which in this dataset was represented only by *Cedecea davisae*.

**Fig. 2.**
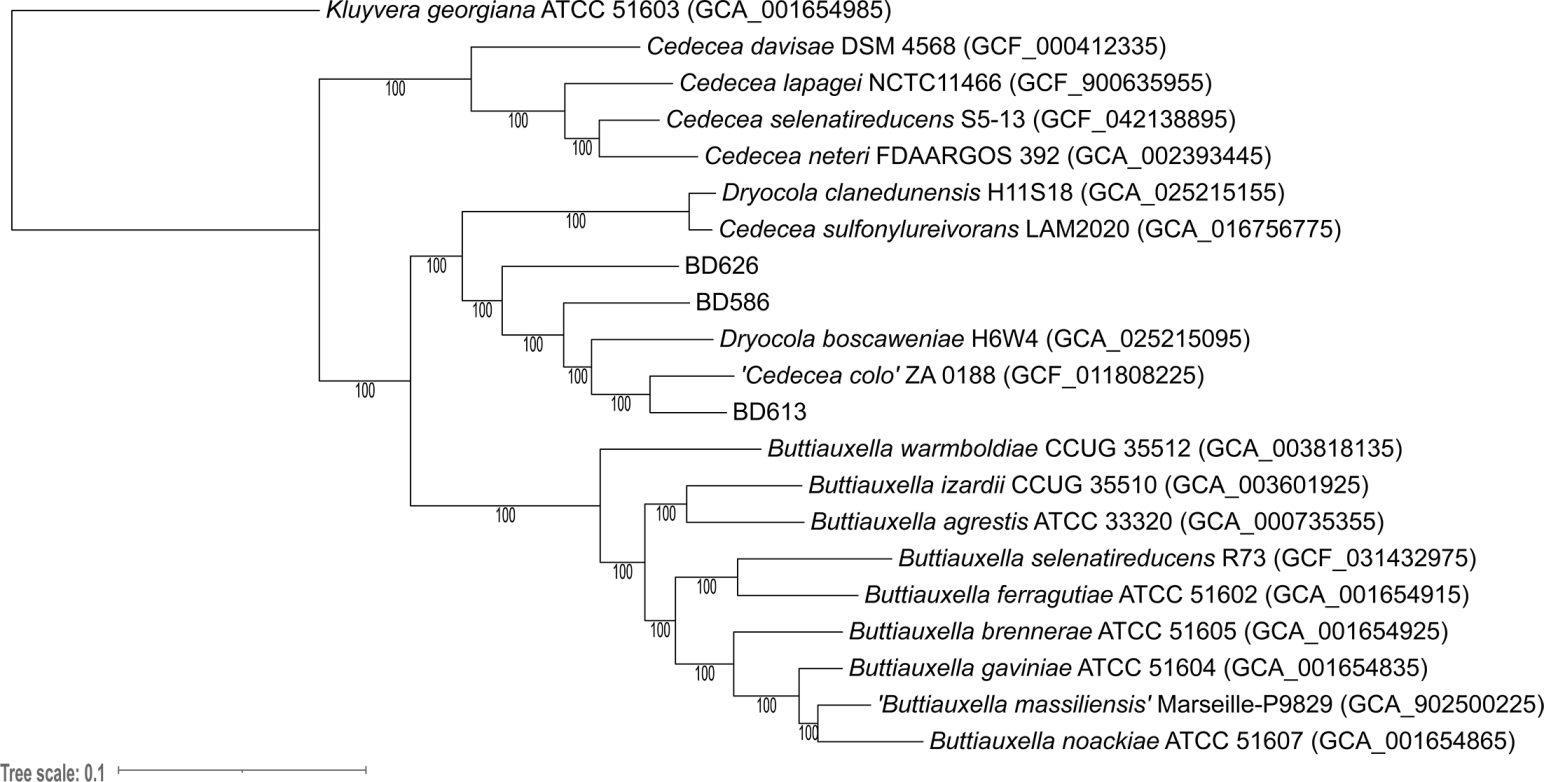
Midpoint rooted phylogenetic tree established using maximum-likelihood methods (IQ-TREE [15]) from core gene alignment (Roary [14]). Node numbers represent ultrafast bootstrap values. *Kluyvera georgiana* served as the outgroup.

To confirm the distinct placement of the three isolates within *Dryocola* and to clarify their relationships with neighbouring genera, a maximum-likelihood tree was constructed based on concatenated core genes. This analysis included all accessible type strains of *Dryocola*, *Cedecea* and *Buttiauxella*, together with BD586^T^, BD613^T^ and BD626^T^. The core gene phylogeny clearly delineated the *Dryocola* clade from the *Buttiauxella* clade, with both sharing a common ancestor with the *Cedecea* clade, consistent with the topology observed in the TYGS-derived GBDP tree (Fig. 2). Within this framework, BD586^T^, BD613^T^ and BD626^T^ clustered with the two described *Dryocola* type strains, supporting their inclusion in this genus. Notably, *C. sulfonylureivorans* also grouped within the *Dryocola* branch rather than with *Cedecea*, indicating that its current taxonomic assignment may need revision. In addition, ‘*C. colo*’ was resolved within the *Dryocola* clade (as previously shown [8], providing further evidence for its reassignment. Taken together, these results corroborate the TYGS analysis, confirm the placement of our three isolates within *Dryocola* and refine the phylogenetic boundaries among *Dryocola*, *Buttiauxella* and *Cedecea*.

### Phenotypic tests

**Fig. 3.**
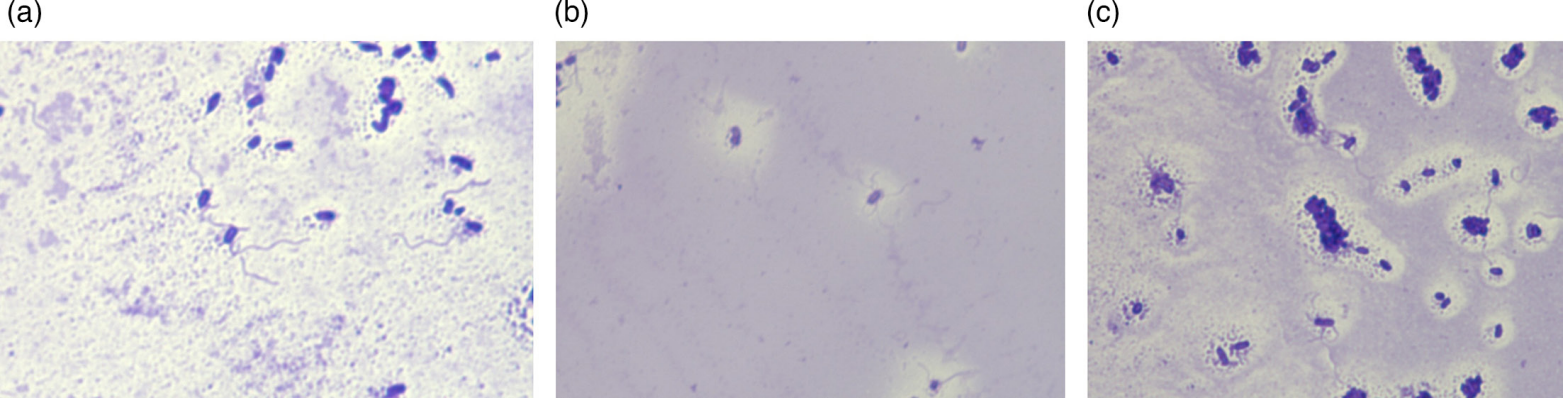
RYU staining of novel *Dryocola* strains revealing peritrichous flagella. Representative light micrographs show cells of (**a**) *D. mayonis* sp. nov. strain BD586^T^, (**b**) *D. sharpae* sp. nov. strain BD613^T^ and (**c**) *D. baronae* sp. nov. strain BD626^T^, each exhibiting peritrichous flagella consistent with the genus description. Images were obtained using a Keyence BZ-X800 microscope at 1,000× total magnification.

All three novel *Dryocola* species grow as white, smooth, opaque, non-haemolytic colonies on tryptic soy agar with 5% sheep blood. All appear as Gram-negative rods by Gram staining. Cells occur singly, in pairs and in short chains, consistent with the genus description of *Dryocola*; BD613^T^ additionally forms small aggregates (Fig. S1). All show motility when cultured in a semi-solid medium with 0.3% (w/v) agar. All are catalase-positive and oxidase-negative. All grow well at 30–37 °C under aerobic conditions and in 5–7% CO_2_ on tryptic soy agar with 5% sheep blood and under anaerobic conditions on CDC anaerobic 5% sheep blood agar (Becton Dickinson). Further, all grow well in 0, 6 and 10% (w/v) NaCl and at pH 7–9, with weak growth at pH 6 and no growth at pH 4–5 or 10. Transmission electron microscopy confirms that all three have a rod-shaped morphology with cell dimensions of ~0.50–0.58×1.19–1.51, 0.43–0.62×1.31–1.55 and 0.38–0.53×1.09–1.19 µm for BD586^T^, BD613^T^ and BD626^T^, respectively (Fig. S1). All strains exhibit peritrichous flagella as revealed by RYU staining and light microscopy at 1,000×magnification, consistent with the genus description of *Dryocola* (Fig. 3).

### Antimicrobial susceptibility assessment

Phenotypic antimicrobial susceptibility testing demonstrated that all three strains were susceptible to several antibiotics, including meropenem, ciprofloxacin and gentamicin (Table S2). BD586^T^ tested intermediate to fosfomycin (applying an *Escherichia coli* breakpoint), while BD613^T^ tested intermediate to cefazolin. BD626^T^ tested resistant to ampicillin, cefazolin and cefdinir (Table S2). BD586^T^ and BD613^T^ tested susceptible to ampicillin, while BD626^T^ tested resistant. This may indicate intra-genus variation in ampicillin susceptibility within *Dryocola*, as resistance was not observed in the type strains reported by Maddock *et al.* [8], but was detected in BD626^T^.

### Chemotaxonomic tests

Biochemical characterization of the three strains was performed using API 50 CH, API ZYM and API 20 E kits. Detailed API test results are provided in Table S3, with key differentiating biochemical characteristics summarized in Table 3. Data for reference strains were obtained from the literature [8, 26]. For API 50 CH, variability among the strains was observed for glycerol, d-adonitol, d-maltose, d-raffinose and potassium 5-ketogluconate. For example, BD626^T^ fermented d-raffinose, whereas BD586^T^ was the only isolate to ferment potassium 5-ketogluconate. Both BD586^T^ and BD626^T^, but not BD613^T^, fermented glycerol and d-maltose, while d-adonitol was fermented by BD586^T^ and BD613^T^ but not BD626^T^. For API 20 E, arginine dihydrolase activity was detected in BD586^T^ and BD626^T^, and acetoin production was observed for BD626^T^ alone. When compared to closely related species, such as *D. boscaweniae*, *D. clanedunensis* and *C. sulfonylureivorans*, the new strains exhibited unique profiles. BD626^T^ fermented d-tagatose, a trait absent in all three related species. Additionally, d-arabitol was fermented by BD586^T^ and BD613^T^, but not *D. clanedunensis* or *C. sulfonylureivorans*. Furthermore, all three strains fermented *N*-acetylglucosamine, which was not observed for *D. boscaweniae* or *D. clanedunensis*. For API ZYM, the three exhibited largely similar profiles, with positive activity for acid phosphatase, naphthol-AS-BI-phosphohydrolase, alkaline phosphatase, esterases (C4, C8) and glycosidases (*β*-galactosidase, *α*-glucosidase, *β*-glucosidase). *β*-glucuronidase activity was detected only in BD586^T^, providing a minor phenotypic distinction from BD613^T^ and BD626^T^ (Table S3). Taken together, the biochemical profiles of all three, including the observed variability in carbohydrate fermentation and enzymatic activities, support their recognition as distinct species.

**Table 3.** Comparison of biochemical profiles of BD586^T^, BD613^T^ and BD626^T^ with other closely related species Strains: 1, *D. mayonis* BD586^T^; 2, *D. sharpae* BD613^T^; 3, *D. baronae* BD626^T^; 4, *D. boscaweniae*; 5, *D. clanedunensis*; 6, *C. sulfonylureivorans*. Symbols: +, positive; −, negative; w, weak positive; v, variable; na, not available. Biochemical data for strains 1–3 were obtained in this study, while data for strains 4–6 were derived from the literature [8,26].

Reaction	*D. mayonis* BD586^T^	*D. sharpae* BD613^T^	*D. baronae* BD626^T^	*D. boscaweniae*	*D. clanedunensis*	*C. sulfonylureivorans*
Fermentation of						
Glycerol	+	−	+	+	v	+
d-Arabinose	−	−	−	−	−	w
d-Adonitol	+	+	−	+	−	w
Methyl-*α*-d-glucopyranoside	−	−	−	+	+	w
*N*-Acetylglucosamine	+	+	+	−	−	+
*Amygdalin	+	+	v	na	na	−
d-Maltose	+	−	+	na	na	+
d-Lactose (bovine)	+	+	+	+	−	w
d-Melibiose	−	−	−	+	−	+
d-Raffinose	−	−	+	+	−	+
Starch (amidon)	−	−	−	na	na	+
d-Lyxose	−	−	−	+	−	−
d-Tagatose	−	−	+	na	na	−
l-Fucose	−	−	−	−	v	+
d-Arabitol	+	+	−	+	−	−
Potassium 2-ketogluconate	+	+	+	+	−	−
Potassium 5-ketogluconate	+	−	−	+	−	−
Voges Proskauer	−	−	+	na	na	−
Enzymatic activity						
Arginine dihydrolase	+	−	+	+	+	+
Ornithine decarboxylase	−	−	−	−	v	na
Utilization of						
Citrate	−	−	−	−	−	+

*Amygdalin was fermented in API 50 CH but not in API 20 E; the result was therefore recorded as variable for *D. baronae* (BD626T).

A detailed comparison of the fatty acid profiles of the novel strains and reference *Dryocola* species is presented in Table 3. The major cellular fatty acids (>10% of total fatty acids) of BD586^T^ were palmitic acid (C_16:0_, 33.6%) and cyclopropane fatty acid (C_17:0_ cyclo, 26.6%). For BD613^T^, the major fatty acids were palmitic acid (C_16:0_, 36.7%) and cyclopropane fatty acid (C_17:0_ cyclo, 25.9%). BD626^T^ was characterized by a higher proportion of myristic acid (C_14:0_, 11.3%) compared to the other strains, with palmitic acid (C_16:0_, 26.6%) and cyclopropane fatty acid (C_17:0_ cyclo, 17.0%) also present as major components. Among the three novel strains, BD626^T^ was notable for its elevated myristic acid (C_14:0_) content, which was not a major component in BD586^T^ or BD613^T^. Comparison with the reference species *D. boscaweniae* and *D. clanedunensis* revealed that palmitic acid (C_16:0_) and cyclopropane fatty acid C_17:0_ cyclo were consistent among the dominant fatty acids across all *Dryocola* strains examined, supporting their role as core chemotaxonomic markers for the genus. However, distinct differences were observed in the relative abundance of myristic acid (C_14:0_), C_19:0_ cyclo *ω*8*c* and minor fatty acids (see Table 4, summed features 2 and 8), underscoring the unique profiles of each species.

**Table 4.** Cellular fatty acid profiles of *D. mayonis* BD586^T^, *D. sharpae* BD613^T^ and *D. baronae* BD626^T^ compared with related *Dryocola* species based on MIDI analysis, with GC-MS clarification

Fatty acid	*D. mayonis* BD586^T^*	*D. sharpae* BD613^T^*	*D. baronae* BD626^T^*	*D. boscaweniae* CCUG 76178*	*D. boscaweniae*†	*D. clanedunensis*†
C_12:0_	6.6	5.9	8.6	7.3	3.9	4.2
C_14:0_	7.4	6.8	11.3	8.7	5.8	6.9
C_15:0_	1.4	1.6	1.4	1.3	na	na
C_16:0_	33.6	36.7	26.6	32.4	34.0	31.3
C_17:0_	1.2	0.7	1.9	1.2	0.4	0.5
C_17:0_ cyclo	26.6 (C_17:0_ cyclo *ω*7*c*)	25.9 (C_17:0_ cyclo *ω*7*c*)	17.0 (C_17:0_ cyclo: 16.6, C_17:1_ *ω*6*c*: 0.4)	20.6 (C_17:0_ cyclo *ω*7*c*)	13.6	12.6
C_19:0_ cyclo *ω*8*c*	5.7 (C_19:0_ cyclo *ω*7*c*)	3.6 (C_19:0_ cyclo *ω*7*c*)	2.0 (C_19:0_ cyclo *ω*7*c*)	5.7 (C_19:0_ cyclo *ω*7*c*)	0.93	2.1
Summed features						
2: C_14:0_ 3OH and/or iso C_16:1_ (GC-MS-resolved components)	8.9 (C_14:0_ 3OH only)	6.9 (C_14:0_ 3OH only)	14.6 (C_14:0_ 3OH only)	10.8 (C_14:0_ 3OH only)	9.3	8.8
3: C_16:1_ *ω*7*c* and/or C_16:1_ *ω*6*c* (GC-MS-resolved components)	2.7 (C_16:1_ *ω*7*c*: 2.6, C_16:1_ *ω*7*t*: 0.1)	4.8 (C_16:1_ *ω*7*c*)	4.8 (C_16:1_ *ω*7*c*: 4.6, C_16:1_ *ω*7*t*: 0.2)	4.4 (C_16:1_ *ω*7*c*: 4.2, C_16:1_ *ω*7*t*: 0.2)	19.9	15.8
5: C_18:2_ *ω*6,9*c* and/or C_18:0_ ante (GC-MS-resolved components)	na (C_18:2_ *ω*6,9*c*: 0.2, C_18:0_ ante nd)	na (C_18:2_ *ω*6,9*c*: 0.2, C_18:0_ ante nd)	na	na (C_18:2_ *ω*6,9*c*: 0.1, C_18:0_ ante nd)	0.7	0.5
8: C_18:1_ *ω*7*c* and/or C_18:1_ *ω*6*c* (GC-MS-resolved components)	2.8 (C_18:1_ *ω*7*c*: 2.5, C_18:1_ *ω*7*t*: 0.3)	3.1 (C_18:1_ *ω*7*c*: 3.0, C_18:1_ *ω*7*t*: 0.1)	4.9 (C_18:1_ *ω*7*c*: 4.2, C_18:1_ *ω*7*t*: 0.7)	3.6 (C_18:1_ *ω*7*c*: 3.3, C_18:1_ *ω*7*t*: 0.3)	11.3	16.0

Fatty acid contents are presented as percentages of total fatty acids. Values for BD586T, BD613T and BD626T are derived from both MIDI/TSBA6 and GC-MS analyses. The cellular fatty acid profile for *D. boscaweniae* was re-analysed in this study using the MIDI/GC-FID method with additional GC-MS confirmation; values for *D. clanedunensis* and literature-based *D. boscaweniae* are taken from previous MIDI-based studies [8]. While the new *D. boscaweniae* CCUG 76178 profile is broadly consistent with published data, some quantitative differences were observed, likely reflecting biological variability and differences in analytical instrumentation and conditions. The new data provide enhanced resolution of summed features and minor components. GC-MS-resolved components of summed features in the new strains and the new *D. boscaweniae* CCUG 76178 profile are shown in parentheses within the same cell. For consistency, C_18:1_ *ω*7*c* values reported individually in the literature are listed under summed feature 8 (C_18:1_ *ω*7*c* and/or C_18:1_ *ω*6*c*), which corresponds to the MIDI definition. nd=Not detected by GC-MS; na=not detected by MIDI (in new strains) or not available in the literature (for reference strains).

*Present study.

†Maddock *et al*. [8].

The polar lipid profiles of BD586^T^, BD613^T^, BD626^T^ and *D. boscaweniae* CCUG 76178 (newly generated in this study) were analysed by two-dimensional silica gel TLC. The number of detected spots per lipid class was determined from TLC images. All shared phosphatidylethanolamine as a common identified component. BD613^T^ and *D. boscaweniae* CCUG 76178 additionally contained diphosphatidylglycerol and phosphatidylglycerol, which were not detected in BD586^T^ or BD626^T^. BD586^T^ had two unidentified glycophospholipids, three unidentified phospholipids, one unidentified aminophospholipid and one unidentified lipid. BD613^T^ had two unidentified glycophospholipids, two unidentified phospholipids, one unidentified aminophospholipid and one unidentified lipid. BD626^T^ exhibited greater diversity in unidentified components, comprising two glycophospholipids, four phospholipids, one aminophospholipid and three lipids. The polar lipid profile of *D. boscaweniae* CCUG 76178 included phosphatidylethanolamine, diphosphatidylglycerol, phosphatidylglycerol, one unidentified aminophospholipid, two unidentified glycophospholipids, one unidentified phospholipid and two unidentified lipids.

In summary, comprehensive data from biochemical, phenotypic and phylogenetic studies indicate that strains BD586^T^, BD613^T^ and BD626^T^ each represent novel *Dryocola* species. The names *Dryocola mayonis* sp. nov., *Dryocola sharpae* sp. nov. and *Dryocola baronae* sp. nov. are proposed for these species, respectively, with each isolate serving as the type strain for its proposed species.

## Description of *Dryocola mayonis* sp. nov.

*Dryocola mayonis* (ma.yo’nis. N.L. gen. n. *mayonis*, pertaining to William J. Mayo, M.D., founder of Mayo Clinic).

Cells are Gram-negative, facultatively anaerobic, motile with peritrichous flagella, rod-shaped (0.50–0.58×1.19–1.51 µm) and occur singly, in pairs and in short chains. Cells are catalase-positive and oxidase-negative. Cells grow at temperatures between 30 and 37 °C, at NaCl concentrations of 0, 6 and 10% (w/v) and at pH 7–9, with weak growth at pH 6. Colonies are white, smooth, opaque, non-haemolytic and 1.5–2.5 mm in diameter on tryptic soy agar with 5% sheep blood. Acid is produced from glycerol, l-arabinose, d-ribose, d-xylose, d-adonitol, d-galactose, d-glucose, d-fructose, d-mannose, l-rhamnose, d-mannitol, *N*-acetylglucosamine, amygdalin, arbutin, aesculin, ferric citrate, salicin, d-cellobiose, d-maltose, d-lactose, d-trehalose, gentiobiose, d-arabitol, potassium gluconate, potassium 2-ketogluconate and potassium 5-ketogluconate. The organism is positive for arginine dihydrolase, alkaline phosphatase, leucine arylamidase, cystine arylamidase, acid phosphatase, naphthol-AS-BI-phosphohydrolase, esterase (C4), esterase lipase (C8), *β*-galactosidase, *α*-glucosidase, *β*-glucosidase and *β*-glucuronidase. The major cellular fatty acids are C_16:0_ and C_17:0_ cyclo. Polar lipids include phosphatidylethanolamine, two unidentified glycophospholipids, one unidentified aminophospholipid, three unidentified phospholipids and an unidentified lipid.

The type strain BD586^T^ (=TSD 474^T^, =NCTC 15089^T^, =DSM 119465^T^) was isolated from a human right knee. The DNA G+C content of the type strain is 56.21 mol%. The GenBank accession number for the complete genome of BD586^T^ is CP150452, and the accession number for its 16S rRNA gene is PQ048012.

## Description of *Dryocola sharpae* sp. nov.

*Dryocola sharpae* (shar’pae. N.L. gen. n. *sharpae*, pertaining to Susan E. Sharp, Ph.D., a medical microbiologist who served as President of the American Society for Microbiology [27]).

Cells are Gram-negative, facultatively anaerobic, motile with peritrichous flagella, rod-shaped (0.43–0.62×1.31–1.55 µm) and occur singly, in pairs and in short chains; cells additionally form small aggregates. Cells are catalase-positive and oxidase-negative. Cells grow at temperatures between 30 and 37 °C, at NaCl concentrations of 0, 6 and 10% (w/v) and at pH 7–9, with weak growth at pH 6. Colonies are white, smooth, opaque, non-haemolytic and 1.5–2.5 mm in diameter on tryptic soy agar with 5% sheep blood. Acid is produced from l-arabinose, d-ribose, d-xylose, d-adonitol, d-galactose, d-glucose, d-fructose, d-mannose, l-rhamnose, d-mannitol, *N*-acetylglucosamine, arbutin, aesculin ferric citrate, salicin, d-cellobiose, d-lactose, d-trehalose, gentiobiose, d-arabitol, potassium gluconate, potassium 2-ketogluconate, amygdalin and arabinose. The organism is positive for alkaline phosphatase, leucine arylamidase, cystine arylamidase, acid phosphatase, naphthol-AS-BI-phosphohydrolase, esterase (C4), esterase lipase (C8), *β*-galactosidase, *α*-glucosidase and *β*-glucosidase. The major cellular fatty acids are C_16:0_ and C_17:0_ cyclo. Polar lipids include phosphatidylethanolamine, diphosphatidylglycerol, phosphatidylglycerol, two unidentified glycophospholipids, one unidentified aminophospholipid, two unidentified phospholipids and one unidentified lipid.

The type strain is BD613^T^ (=TSD 475^T^, =NCTC 15090^T^, =DSM 119466^T^), which was isolated from a human right foot. The DNA G+C content of the type strain is 54.55 mol%. The GenBank accession number for the complete genome of BD613^T^ is CP149994, and the accession number for its 16S rRNA gene is PQ048013.

## Description of *Dryocola baronae* sp. nov.

*Dryocola baronae* (ba.ro’nae. N.L. gen. n. *baronae*, pertaining to Ellen Jo Baron, Ph.D., a medical microbiologist who advanced rapid diagnostic methods for infectious diseases [28]).

Cells are Gram-negative, facultatively anaerobic, motile with peritrichous flagella, rod-shaped (0.38–0.53×1.09–1.19 µm) and occur singly, in pairs and in short chains. Cells are catalase-positive and oxidase-negative. The bacterium grows at temperatures between 30 and 37 °C, at NaCl concentrations of 0, 6 and 10% (w/v) and at pH 7–9, with weak growth at pH 6. Colonies are white, smooth, opaque, non-haemolytic and 1.5–2.5 mm in diameter on tryptic soy agar with 5% sheep blood. Acid is produced from glycerol, l-arabinose, d-ribose, d-xylose, d-galactose, d-glucose, d-fructose, d-mannose, l-rhamnose, d-mannitol, *N*-acetylglucosamine, arbutin, aesculin ferric citrate, salicin, d-cellobiose, d-maltose, d-lactose, d-trehalose, d-raffinose, gentiobiose, d-tagatose, potassium gluconate, potassium 2-ketogluconate, amygdalin (variable) and arabinose. The organism is positive for arginine dihydrolase, acetoin production, alkaline phosphatase, leucine arylamidase, cystine arylamidase, acid phosphatase, naphthol-AS-BI-phosphohydrolase, esterase (C4), esterase lipase (C8), *β*-galactosidase, *α*-glucosidase, *β*-glucosidase and *β*-glucuronidase. The major cellular fatty acids are C_14:0_, C_16:0_ and C_17:0_ cyclo. Polar lipids include phosphatidylethanolamine, two unidentified glycophospholipids, an unidentified aminophospholipid, four unidentified phospholipids and three unidentified lipids.

The type strain is BD626^T^ (=TSD 476^T^, =NCTC 15091^T^, =DSM 119479^T^), isolated from a human knee. The DNA G+C content of the type strain is 55.85 mol%. The GenBank accession number for the complete genome of BD626^T^ is JBINNY000000000, and the accession number for its 16S rRNA gene is PQ048222.

## Emended description of the genus *Dryocola*

*Dryocola* (Gr. fem. n. *drys*, an oak; L. suff. –cola, inhabitant; N.L. masc. n. *Dryocola*, an inhabitant of oaks).

Cells are Gram-negative rods, measuring 0.38–0.62×1.09–2.1 µm, facultatively anaerobic, oxidase-negative and catalase-positive. They occur singly, in pairs and in short chains; some species (e.g., *D. sharpae*) may also form small aggregates. Cells are motile with peritrichous flagella. Colonies on tryptic soy agar are smooth, white to cream-coloured, opaque and non-haemolytic, occasionally with a darker convex centre and uneven margins. The major fatty acids are C_16:0_ and C_17:0_ cyclo, with minor components varying among species. The genomic DNA G+C content ranges from 53.0 to 56.2 mol%. The type species is *D. boscaweniae* [8].

## Conclusion

This study describes three novel species of the genus *Dryocola*, namely *D. mayonis* sp. nov., *D. sharpae* sp. nov. and *D. baronae* sp. nov., isolated from human clinical specimens. A polyphasic approach integrating whole-genome comparisons, phylogenomic analyses, phenotypic testing and chemotaxonomic characterization established their novelty and placement within *Dryocola*. The addition of these species expands the known diversity of the genus and demonstrates its presence in human-associated environments. Notably, the genomic G+C contents of the three strains (54.5–56.2 mol%) extend beyond the range previously reported for *Dryocola* (53.0–53.9 mol%). Accordingly, we formally emend the description of the genus to broaden the reported G+C content, refine cell size ranges, clarify cellular arrangement (singly, pairs and short chains, with small aggregates in some species), expand colony morphology and define C_16:0_ and C_17:0_ cyclo as the major fatty acids. These findings, together with evidence that *C. sulfonylureivorans* and ‘*C. colo*’ cluster within *Dryocola*, highlight the need for a unified re-evaluation to properly define and delimit the genus.

## Supplementary material

10.1099/ijsem.0.006986 Uncited Supplementary Material 1.

## Abbreviations

ANI: average nucleotide identity
dDDH: digital DNA–DNA hybridization
GBDP: genome blast distance phylogeny
ICNP: International Code of Nomenclature of Prokaryotes
MALDI-ToF: matrix-assisted laser desorption ionization-time-of-flight
SGC: SmartGene IDNS™ Centroids
TYGS: type strain genome server
