# Supplementary material for: Description of three new Dryocola species: Dryocola mayonis sp. nov., Dryocola sharpae sp. nov. and Dryocola baronae sp. nov., isolated from human clinical samples
Source: Int J Syst Evol Microbiol. 2025 Dec 2;75(12):006986. doi: 10.1099/ijsem.0.006986 (PMC12671600; doi:10.1099/ijsem.0.006986)
Supplement: Uncited Supplementary Material 1. [file ijsem-75-06986-s001.pdf]

Supplementary Files for Description of Three New *Dryocola* species; *Dryocola mayonis* sp. nov., *Dryocola sharpae* sp. nov., and *Dryocola baronae* sp. nov., Isolated from Human Clinical Samples

Hamidu T. Mohammed,[a] Christina A. Koscianski,[a] Garrett G. Gordy,[a]

Stephen Johnson,[b] Robin Patel [a,c*]

[a]Division of Clinical Microbiology, Department of Laboratory Medicine and Pathology, Mayo Clinic, Rochester, MN 55905, USA

[b]Department of Quantitative Health Sciences, Mayo Clinic, Rochester, MN 55905, USA

[c]Division of Public Health, Infectious Diseases, and Occupational Medicine, Department of Medicine, Mayo Clinic, Rochester, MN 55905, USA

***Corresponding author**

Robin Patel, M.D.

Division of Clinical Microbiology, Mayo Clinic

200 First Street SW, Rochester, MN 55905

Phone - 507-538-0579

email: patel.robin@mayo.edu

GenBank Accession Numbers

Dryocola mayonis sp. nov. strain BD586^T; complete genome: CP150452, 16S rRNA gene: PQ048012

Dryocola sharpae sp. nov. strain BD613^T; complete genome: CP149994, 16S rRNA gene: PQ048013

Dryocola baronae sp. nov. strain BD626^T; complete genome: JBINNY000000000, 16S rRNA gene: PQ048222

This supplementary material accompanies the manuscript (Description of Three New *Dryocola* species; *Dryocola mayonis* sp. nov.*, Dryocola sharpae* sp. nov.*,* and *Dryocola baronae* sp. nov., Isolated from Human Clinical Samples) submitted to the International Journal of Systematic and Evolutionary Microbiology (IJSEM).

Manuscript reference number: **Manuscript IJSEM-D-25-00398**

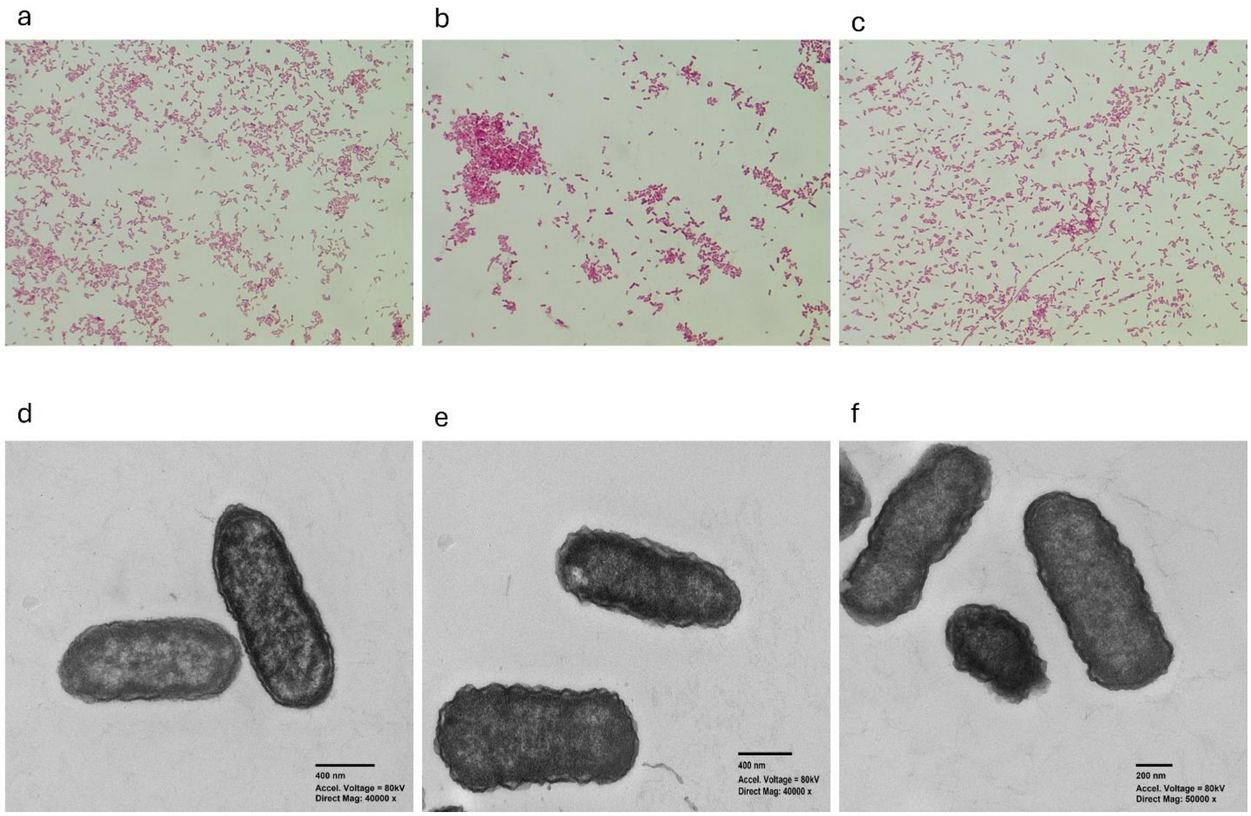

Supplementary Figure S1. Gram-stain and transmission electron micrographs of the three novel *Dryocola* strains.

Gram-stained images of (a) *Dryocola mayonis* BD586^{T}, (b) *Dryocola sharpae* BD613^{T}, and (c) *Dryocola baronae* BD626^{T} show Gram-negative rods. All three strains occur singly, in pairs, and in short chains; BD613^{T} additionally forms small aggregates. Transmission electron micrographs of (d) BD586^{T}, (e) BD613^{T}, and (f) BD626^{T} reveal rod-shaped morphology with observed dimensions of 1.19–1.51 × 0.50–0.58 µm, 1.31–1.55 × 0.43–0.62 µm, and 1.09–1.19 × 0.38–0.53 µm, respectively (N = 2 cells per strain, multiple line profiles measured per dimension).

Supplementary Table S1. Comparison of average nucleotide identify (ANI) and results of digital DNA-DNA hybridization (dDDH) between *Dryocola mayonis* BD586^T, *Dryocola sharpae* BD613^T, *Dryocola baronae* BD626^T and related species. Related strains were identified using TYGS [1, 2]. dDDH values are from the TYGS report [3]; ANI values were calculated using FastANI [4].

	ANI			dDDH		
Strains	BD586^T	BD613^T	BD626^T	BD586^T	BD613^T	BD626^T
BD586^T	100	88.28	86.37	NA	34.6	30.7
BD613^T	88.35	100	85.66	34.6	NA	29.8
BD626^T	86.29	85.76	100	30.7	29.8	NA
Pantoea ananatis LMG 2665	78.35	78.40	78.37	19.9	20.2	20.6
Cronobacter sakazakii NBRC 102416	80.84	80.84	80.70	21.6	21.7	21.6
Buttiauxella agrestis ATCC 33320	81.95	81.99	82.19	23.3	23.5	23.5
Kluyvera cryocrescens NBRC 102467	79.92	79.93	80.00	21.2	21.3	21
Pantoea agglomerans NBRC 102470	78.55	78.47	78.58	20	20.2	20.1
Buttiauxella gaviniae ATCC 51604	81.63	81.69	82.00	22.9	23.2	23.4
Buttiauxella noackiae ATCC 51607	81.90	81.77	82.11	22.9	23	23.5
Buttiauxella ferragutiae ATCC 51602	81.92	81.66	82.11	23.1	23.2	23.5
Buttiauxella brennerae ATCC 51605	81.73	81.82	82.07	23.2	23.3	23.6
Kluyvera georgiana ATCC 51603	80.30	80.93	80.53	21.5	22.7	21.6
Lelliottia amnigena DSM 4486	80.07	79.97	80.13	20.5	20.5	20.9
Mixta gaviniae DSM 22758	79.37	80.47	79.07	20.8	22.6	20.8
Buttiauxella izardii CCUG 35510	82.22	82.24	82.55	23.7	23.9	24.1
Buttiauxella warmboldiae CCUG 35512	82.66	82.68	82.83	24.3	24.2	24.6
Klebsiella trevisanii DSM 2688	80.36	80.33	80.26	21.2	21.5	21.1
Enterobacter dykesii E1T	80.83	80.64	80.95	21.1	21.1	21.4
'Cedecea sulfonylureivorans' LAM2020	84.25	84.15	84.56	27	26.6	27
Dryocola boscaweniae H6W4	88.62	88.64	85.73	35.2	35.8	29.5
Dryocola clanedunensis H11S18	84.34	84.07	84.64	26.7	26.6	26.8
Silvania confinis H4N4	80.47	80.24	80.57	20.8	20.9	21.1
Enterobacter nematophilus E-TC7	80.98	80.83	81.04	21.1	21.1	21.4
Enterobacter pseudoroggenkampii' 155092	80.66	80.62	80.91	21.1	21.1	21.5
'Enterobacter pasteurii' A-8	80.87	80.62	81.06	21.3	21.1	21.5
'Buttiauxella massiliensis' Marseille-P9829	81.75	81.90	81.88	22.9	23.2	23.2
Cedecea davisae DSM 4568	82.18	82.04	82.31	23.3	23.2	23.7
Siccibacter turicensis LMG 23730	80.79	80.68	80.93	21.5	21.3	21.8
Enterobacter quasiroggenkampii WCHECL1060 T	80.71	80.75	80.87	21.1	21.2	21.4
Enterobacter hormaechei subsp. *oharae* DSM 16687	80.80	80.81	81.13	21.4	21.5	21.8
Enterobacter hormaechei subsp. *steigerwaltii* DSM 16691	80.73	80.58	80.89	21.3	21.2	21.7
Enterobacter quasimori 90044	80.73	80.63	81.09	21.1	21.1	21.4

Supplementary Table S2. Results of antimicrobial susceptibility testing. MIC, minimum inhibitory concentration; S, susceptible; I, intermediate; R, resistant.

Antibiotic	BD586^{T}		BD613^{T}		BD626^{T}	
	MIC (µg/mL)	S/I/R*	MIC (µg/mL)	S/I/R*	MIC (µg/mL)	S/I/R*
**Ampicillin**	2	S	2	S	>16	R
**Ampicillin-sulbactam**	≤8/4	S	≤8/4	S	16/8	I
**Meropenem**	≤0.12	S	≤0.12	S	≤0.12	S
**Ertapenem**	≤0.25	S	≤0.25	S	≤0.25	S
**Piperacillin-tazobactam**	≤8/4	S	≤8/4	S	≤8/4	S
**Ciprofloxacin**	≤0.25	S	≤0.25	S	≤0.25	S
**Levofloxacin**	≤0.5	S	≤0.5	S	≤0.5	S
**Cefazolin**	≤2	S	4	I	>16	R
**Ceftriaxone**	≤1	S	≤1	S	≤1	S
**Ceftazidime**	≤4	S	≤4	S	≤4	S
**Cefepime**	≤2	S	≤2	S	≤2	S
**Cefdinir**	≤1	S	≤1	S	>2	R
**Amikacin**	≤4	S	≤4	S	≤4	S
**Gentamicin**	≤1	S	≤1	S	≤1	S
**Tobramycin**	≤1	S	≤1	S	≤1	S
**Aztreonam**	≤4	S	≤4	S	≤4	S
**Trimethoprim-sulfamethoxazole**	≤0.5/9.5	S	≤0.5/9.5	S	≤o.5/9.5	S
**Nitrofurantoin (urine only)****	64	I	64	I	≤32	S
**Fosfomycin*****	>128		≤64		128	
**Colistin**	≤2	I	≤2	I	≤2	S
**Ceftazidime-avibactam**	≤0.12/4	S	≤0.12/4	S	2/4	S
**Ceftolozane-tazobactam**	≤0.25/4	S	≤0.25/4	S	2/4	S
**Meropenem-vaborbactam**	0.12/8	S	0.12/8	S	0.12/8	S
**Minocycline**	≤4	S	≤4	S	≤4	S

* Enterobacterales breakpoints from M100 35th edition applied [5].

**Breakpoint is for urinary isolates only.

***Fosfomycin breakpoint is for *Escherichia coli* only ≤64 S, 128 I, ≥256 R

Supplementary Table S3. Carbohydrate fermentation and enzymatic activity profiles of *Dryocola mayonis* BD586^T, *Dryocola sharpae* BD613^T, and *Dryocola baronae* BD626^T as determined by API 50 CH, API 20 E, and API ZYM tests.

TUBE	TESTS	BD586^T	BD613^T	BD626^T
API 50 CH				
0	Control	-	-	-
1	Glycerol	+	+	+
2	Erythritol	-	-	-
3	D-Arabinose	-	-	-
4	L-Arabinose	+	+	+
5	D-Ribose	+	+	+
6	D-Xylose	+	+	+
7	L-Xylose	-	-	-
8	D-Adonitol	+	+	-
9	Methyl-ß-D-xylopyranoside	-	-	-
10	D-Galactose	+	+	+
11	D-Glucose	+	+	+
12	D-Fructose	+	+	+
13	D-Mannose	+	+	+
14	L-Sorbose	-	-	-
15	L-Rhamnose	+	+	+
16	Dulcitol	-	-	-
17	Inositol	-	-	-
18	D-Mannitol	+	+	+
19	D-Sorbitol	-	-	-
20	Methyl-α-D-mannopyranoside	-	-	-
21	Methyl-α-D-glucopyranoside	-	-	-
22	N-Acetylglucosamine	+	+	+
23	Amygdalin	+	+	V
24	Arbutin	+	+	+
25	Esculin ferric citrate	+	+	+
26	Salicin	+	+	+
27	D-Cellobiose	+	+	+
28	D-Maltose	+	+	+
29	D-Lactose (bovine origin)	+	+	+

30	D-Melibiose	-	-	-
31	D-Saccharose (sucrose)	-	-	-
32	D-Trehalose	+	+	+
33	Inulin	-	-	-
34	D-Melezitose	-	-	-
35	D-Raffinose	-	-	+
36	Starch	-	-	-
37	Glycogen	-	-	-
38	Xylitol	-	-	-
39	Gentiobiose	+	+	+
40	D-Turanose	-	-	-
41	D-Lyxose	-	-	-
42	D-Tagatose	-	-	+
43	D-Fucose	-	-	-
44	L-Fucose	-	-	-
45	D-Arabitol	+	+	-
46	L-Arabitol	-	-	-
47	Potassium gluconate	+	+	+
48	Potassium 2-ketogluconate	+	+	+
49	Potassium 5-ketogluconate	+	-	-
API 20 E				
1	ϐ-Galactosidase (Ortho nitrophenyl-ϐD-galactopyranosidase)	-	-	-
2	Arginine dihydrolase	+	-	+
3	Lysine decarboxylase	-	-	-
4	Ornithine decarboxylase	-	-	-
5	Citrate utilization	-	-	-
6	H_2S production	-	-	-
7	Urease	-	-	-
8	Tryptophan deaminase	-	-	-
9	Indole production	-	-	-
10	Acetoin production (Voges Proskauer)	+	+	+
11	Gelatinase	-	-	-
12	D-Glucose (fermentation - oxidation)	+	+	+
13	D-Mannitol (fermentation - oxidation)	+	+	+
14	Inositol (fermentation - oxidation)	-	-	-
15	D-Sorbitol (fermentation - oxidation)	-	-	-

16	L-Rhamnose (fermentation - oxidation)	+	+	+
17	D-Saccharose (fermentation - oxidation)	-	-	-
18	D-Melibiose (fermentation - oxidation)	-	-	-
19	Amygdalin (fermentation - oxidation)	+	+	V
20	Arabinose (fermentation - oxidation)	+	+	+
21	OXIDASE	-	-	-
API ZYM				
1	Control	-	-	-
2	Alkaline phosphatase	+	+	+
3	Esterase (C 4)	+	+	+
4	Esterase Lipase (C 8)	+	+	+
5	Lipase (C 14)	-	-	-
6	Leucine arylamidase	+	+	+
7	Valine arylamidase	-	-	-
8	Cystine arylamidase	+	+	+
9	Trypsin	-	-	-
10	α-chymotrypsin	-	-	-
11	Acid phosphatase	+	+	+
12	Naphthol-AS-BI-phosphohydrolase	+	+	+
13	α-galactosidase	-	-	-
14	β-galactosidase	+	+	+
15	β-glucuronidase	+	-	-
16	α-glucosidase	+	+	+
17	β-glucosidase	+	+	+
18	*N*-acetyl-β-glucosaminidase	-	-	-
19	α-mannosidase	-	-	-
20	α-fucosidase	-	-	-

References

1. **Meier-Kolthoff JP, Göker M**. TYGS is an automated high-throughput platform for state-of-the-art genome-based taxonomy. *Nat Commun* 2019;10:2182.

2. **Meier-Kolthoff JP, Carbasse JS, Peinado-Olarte RL, Göker M**. TYGS and LPSN: a database tandem for fast and reliable genome-based classification and nomenclature of prokaryotes. *Nucleic Acids Res* 2022;50:D801--D807.

3. **Auch AF, von Jan M, Klenk H-P, Göker M**. Digital DNA-DNA hybridization for microbial species delineation by means of genome-to-genome sequence comparison. *Stand Genomic Sci* 2010;2:117–134.

4. **Seemann T, Klötzl F, Page AJ**. Pairwise SNP distance matrix from a FASTA sequence alignment. *Zenodo https://doi org/105281/zenodo*;1411986.

5. Clinical and Laboratory Standards Institute (CLSI) 2025. Performance Standards for Antimicrobial Susceptibility Testing. CLSI supplement M100. 35th ed. Wayne, PA.: Clinical and Laboratory Standards Institute.
